# The Dissemination of Parent–Child Interaction Therapy in West Virginia during the Opioid Epidemic and COVID-19 Pandemic: A Qualitative Study

**DOI:** 10.3390/ijerph192215085

**Published:** 2022-11-16

**Authors:** Lindsay R. Druskin, Robin C. Han, Sharon T. Phillips, Erinn J. Victory, Emily Aman, Jennifer Tiano, Jocelyn Stokes, Cheryl B. McNeil

**Affiliations:** 1 Department of Psychology, West Virginia University, Morgantown, WV 26506, USA; 2 Department of Psychology, Marshall University, Huntington, WV 25755, USA; 3 Department Behavioral Medicine and Psychiatry, School of Medicine Eastern Division, West Virginia University, Martinsburg, WV 25401, USA; 4 Department of Psychiatry, University of Florida, Gainesville, FL 32610, USA

**Keywords:** PCIT, Parent–Child Interaction Therapy, rural populations, opioid crisis, dissemination, implementation

## Abstract

The devastating impact of the opioid crisis on children and families in West Virginia was compounded by the COVID-19 pandemic and brought to light the critical need for greater mental health services and providers in the state. Parent–Child Interaction Therapy (PCIT) is an evidence-based treatment for child externalizing symptoms that teaches parents positive and appropriate strategies to manage child behaviors. The current qualitative study details barriers and facilitators to disseminating and implementing PCIT with opioid-impacted families across West Virginia during the COVID-19 pandemic. Therapists (*n* = 34) who participated in PCIT training and consultation through a State Opioid Response grant were asked to provide data about their experiences with PCIT training, consultation, and implementation. Almost all therapists (91%) reported barriers to telehealth PCIT (e.g., poor internet connection, unpredictability of sessions). Nearly half of therapists’ cases (45%) were impacted directly by parental substance use. Qualitative findings about the impact of telehealth and opioid use on PCIT implementation are presented. The dissemination and implementation of PCIT in a state greatly impacted by poor telehealth capacity and the opioid epidemic differed from the implementation of PCIT training and treatment delivery in other states, highlighting the critical importance of exploring implementation factors in rural settings.

## 1. Introduction

Over the past two decades, the number of drug overdose deaths in the United States has quadrupled, with the majority of overdose deaths involving opioids [1,2]. West Virginia has been deemed the epicenter of the opioid epidemic as it is ranked among the states with the highest levels of opioid overdoses [1]. In 2020, the opioid overdose rate in West Virginia was three times more than the national average (70 deaths compared to 21.4 deaths per 100,000 people) [1].

The impact of the opioid epidemic is not limited to those who use opioids; there is a lesser known but all too devastating impact on children and families. Parental substance use, which is considered an adverse childhood experience (ACE) [3,4,5,6], places children at an increased risk of experiencing toxic stress, attachment issues, behavior problems, and child welfare involvement (e.g., placement in foster care) [7,8,9]. Recent reports show drug overdose rates are largely associated with child foster care entries in Appalachia [8], and according to data, over 34,000 children in West Virginia have been placed in the primary custody of kinship caregivers such as grandparents [10]. Thus, the effects of the opioid crisis on children and families in West Virginia are evident, as the state is ranked among the highest in the nation for rates of child abuse and neglect and child foster care entries [11,12].

Situated in rural Appalachia, West Virginia has a unique set of challenges to accessing mental health services, including provider shortages, geographic barriers, and poor transportation infrastructure [13,14,15]. The limited service accessibility and availability are compounded by the issue of service affordability, especially considering that West Virginia has the sixth highest poverty rate in the country as of 2020 [16]. During the COVID-19 pandemic, many individuals began receiving healthcare services via telehealth [17]. While there is limited research on telehealth services in rural Appalachia, a recent study found that individuals from West Virginia and surrounding areas were 20% more likely to complete a telehealth primary care appointment than an in-person appointment [18]. However, for many residing in rural areas, telehealth services are not a reliable option due to insufficient internet access [19].

### 1.1. The Need for Parent–Child Interaction Therapy in West Virginia

Due to the extensive problems that the COVID-19 pandemic and the opioid epidemic have highlighted, there is a clear need for evidence-based interventions to be disseminated to families in West Virginia. Parent–Child Interaction Therapy (PCIT) is an intervention designed to reduce child behavior problems by strengthening the caregiver-child relationship and providing families with effective behavior management skills [20]. PCIT is based in operant learning theory and structured as a two-stage model emphasizing both nurturance and limit setting, as outlined in Baumrind’s description of the authoritative parenting style [20,21]. PCIT has extensive evidence for its effectiveness in reducing child externalizing and internalizing symptoms as well as parenting stress [22,23]; thus it would be well suited to address behavioral problems that families exposed to substance use experience. Although traditional PCIT is designed for children ages 2 to 7 years, there are adaptations that extend the age range down to 12 months and up to 10 years of age [24,25] making it a suitable option for widespread dissemination.

Previous research examining the dissemination and implementation of PCIT indicates that PCIT can be disseminated to community clinicians with high fidelity [26]. Findings from these PCIT dissemination studies suggest that PCIT is acceptable to clinicians and that the intervention leads to positive outcomes for child behavior [26,27]. However, there are significant barriers to these clinicians being able to successfully implement PCIT in their community-based clinics, including limited time, the training requirements of becoming a certified PCIT therapist, need for more experiential learning, and attitudes towards evidence-based practices [27,28,29]. Due to the COVID-19 pandemic, some PCIT dissemination and implementation work transitioned to online delivery [30,31]. Prior to the pandemic, Funderburk and colleagues [32] suggested that the use of videoconferencing technology could address some limitations of in-person dissemination including allowing for more involved consultation. PCIT implements a unique set-up that seamlessly adapts to the telehealth format, as much of the session is conducted with the therapist and family in different rooms [31,33]. Garcia and colleagues [30] successfully disseminated and implemented virtual PCIT and found that families involved in the implementation project significantly improved in regard to caregiver stress and child behavior problems. However, such findings are in the context of an urban setting, and thus more work needs to be done to expand implementation efforts to additional populations, especially ones that may experience increased barriers to accessing evidence-based mental health care.

### 1.2. The State Opioid Response Grant

The purpose of the PCIT State Opioid Response Medical Services Grant, hereby referred to as the SOR Grant, was to increase the availability and sustainability of PCIT across West Virginia to ultimately address the problem of childhood emotional and behavioral issues resulting from the opioid crisis. The dissemination project entailed two primary aims: (1) to increase the availability of PCIT in West Virginia by training mental health professionals to deliver this intervention to families in their respective catchment areas, and (2) to maintain the sustainability of PCIT services by training therapists to become certified trainers of the intervention so that they could teach PCIT to other providers in their agencies. The dissemination strategy employed in the current grant utilized a combination of two training methods, the Learning Collaborative Model (i.e., including multiple levels of professionals, such as clinical administrators and clinicians, when implementing training in an organization), and the Cascading Model (i.e., training one provider at an agency who can then train other provders); also known as the “train the trainer” model) [34]. A training model was designed and delivered over the course of three years (July 2019 to June 2022). As the COVID-19 crisis developed in Spring 2020, later cohorts of therapists received virtual PCIT training and consultation entirely through the Zoom platform.

### 1.3. The Current Study

The current study utilized a qualitative content analysis strategy to examine the ways in which the dissemination of PCIT in the state of West Virginia was impacted by the opioid epidemic and COVID-19 pandemic. The aims of the current study were to determine how the opioid crisis and COVID-19 pandemic affected (1) therapist training experiences and (2) the provision of PCIT services to families across the state. It was hypothesized that the pandemic would negatively impact therapists’ recruitment of PCIT cases and completion of certification requirements. Additionally, it was hypothesized that the service delivery of PCIT during the COVID-19 pandemic would be challenging for families given the lack of technological infrastructure in the state of West Virginia [16]. We hypothesized that these challenges would be heightened for families directly and indirectly affected by the opioid crisis due to the added layer of complexity typical of such cases, such as attachment issues, child welfare involvement, and grandparent caregivers [7,8,9].

We present qualitative data to describe therapist experiences with a mixed in-person and virtual PCIT training procedure and implementation outcomes following this training. Specifically, we describe (1) the therapist participation in training (e.g., completion rate, consultation participation, and PCIT certification status), (2) strengths and weaknesses of in-person vs. virtually delivered training and consultation, (3) the impact of the opioid crisis and COVID-19 pandemic on service delivery and client retention, and (4) unique factors affecting PCIT services within a client population embedded in West Virginia’s current dual-crisis climate. The current study received approval from the Institutional Review Board of the research team’s university (protocol code 2107354044).

## 2. Materials and Methods

### 2.1. Trainers

Three clinical psychologists conducted all training activities and consultation calls. PCIT International, the professional organization that oversees PCIT training and certification, delineates three levels of trainers: (1) Within-Agency Trainers (certified to train providers within their organizations or practices), (2) Regional Trainers (certified to train and consult with providers within and outside of their own agency, restricted by location), and (3) Global Trainers (highest trainer status, certified to train and consult with therapists in any location). Two of the trainers on the SOR Grant were Within-Agency Trainers working toward Regional Trainer certification, and the third trainer was a Global Trainer.

### 2.2. Trainees

#### 2.2.1. Recruitment

Email advertisements and word of mouth were used to recruit therapists for participation in the training activities. Interested individuals completed an application form in which they provided information about their typical caseload and agency support to deliver PCIT. Three cohorts of training (total *n* = 80) occurred from July 2019 to June 2022.

#### 2.2.2. Description of Therapists

The majority of therapists across the three cohorts held master’s degrees in psychology, social work, behavior analysis, or counseling (71%). The remaining therapists were doctoral-level clinical psychologists (24%), psychiatrists (4%), and graduate students enrolled in master’s programs (3%). Therapists served families across several agency settings, including community-based mental health centers (31%), private practice (25%), outpatient behavioral health clinics (19%), university-based training clinics (14%), and child advocacy centers (10%). All but one therapist (97%) who were admitted into the SOR training cohorts attended a 40 h PCIT training. The majority of therapists (61%) attended greater than 75% of their consultation call series.

### 2.3. Training Description

#### 2.3.1. Training of PCIT Therapists

Masters- and doctoral-level therapists across West Virginia were recruited to participate in a 40 h workshop in PCIT led by a certified trainer. Upon completion of the training, therapists received equipment (e.g., baby monitors, bluetooth earpieces, toys, assessments) to implement PCIT in their respective agencies. Additionally, therapists received biweekly group consultation for one year. These consultation calls were crucial to the training model, as they were intended to provide therapists with clinical and technical support, thereby increasing the likelihood that therapists would continue delivering the intervention [35,36]. Per PCIT International guidelines, therapists needed to successfully graduate two PCIT cases and receive a fidelity check of their Child-Directed Interaction (CDI) and Parent-Directed Interaction (PDI) skills for certification purposes.

#### 2.3.2. Training of Within-Agency Trainers and Regional Trainers

A portion of the budget during the second year of the grant was dedicated to increasing the sustainability of PCIT services across the state by training certified PCIT therapists to become Within-Agency Trainers (WAT) and Regional Trainers. For WAT certification, therapists participated in an 8 h workshop, monthly consultation calls over the course of one year, and identified one masters-level therapist in their organization to train in PCIT using a co-therapy model. Two of three psychologists involved in the SOR grant sought Regional Trainer certification status, requiring them to achieve several higher-level training and supervision competencies.

### 2.4. Participants Included in Qualitative Analysis

All therapists who received training were asked to provide follow-up data on their experiences and PCIT cases obtained following training. At the time of data collection, 31% of trainees were certified as PCIT therapists. A total of 34 individuals responded to provide follow-up data on their training experiences for the current study. Therapists (*n* = 34) included doctoral level (37.1%) and masters level (57.1%) providers, as well as 5.7% doctoral student trainees. The majority of the sample (74.3%) were licensed providers in West Virginia, with an additional 17.2% currently seeking licensure. On average, therapist providers had practiced in the mental health field for 10.47 years (*SD* = 8.03). Additionally, therapists in the sample offered a variety of options for clients to pay for therapeutic services, including self-pay, insurance, sliding scale fees, and services free of charge. Three of these therapists had previously sought PCIT Certification and were seeking Within-Agency Trainer status, allowing them to train other therapists at their own agency or program. Additional participant characteristics are presented in Table 1. Of these 34 therapists, 22 were trained solely in an in-person format, 5 were trained entirely in a virtual format, and 6 therapists were trained using a hybrid format.

### 2.5. Procedure

Following the completion of training and a year-long, bi-weekly consultation series, therapists were contacted via email to participate in an interview about their involvement in PCIT training and service delivery. After participants were scheduled for an interview, they received a Zoom link, and two study personnel conducted a structured interview. Interview questions covered information about the participants’ thoughts about their training experience (e.g., “What could be improved about the trainings you attended?”), barriers to implementing PCIT after training (e.g., “What barriers did you encounter in obtaining and completing PCIT cases?”), and information about clients they used PCIT with (e.g., how many PCIT cases dropped out, graduated).

Twelve trained study personnel conducted interviews to collect data about therapist experiences with and perceptions of PCIT training and implementation. Training consisted of attending an hour-long didactic with graduate students experienced with qualitative interviews and an observation period where graduate students watched study personnel conducting their first interviews. Training included reviewing and rehearsing the interview script and general interview etiquette. The codebook for qualitative analysis was developed using conventional and inductive qualitative analyses, which involved study authors reviewing all interviews and identifying the key content [37,38]. Each interview was transcribed verbatim and segments of text were coded manually, without the use of a software. In line with a directed content analysis [37], codes were defined before coding to align with the semi-structured interview using a deductive process [9,27,28,29,30]. During the coding process, codes were refined throughout the process with consensus by the research team to capture important data-driven themes identified by study personnel using an inductive process. Study personnel met weekly for the duration of the coding process to discuss the clarity and scope of coding categories, thus ensuring the identification of clearly defined themes. Each transcript was randomly assigned to two study personnel for independently parallel coding to ensure thorough and comprehensive thematic analysis. After coding each transcript, the personnel met to discuss their codes; coder disagreements were discussed until consensus agreement was reached. At times, more than one code was applied to a single text segment when it applied to multiple qualitative themes.

## 3. Results

Thirty-four interviews, broken into 3192 text segments, were coded using principles of thematic analysis. A total of 32 unique codes were identified, as well as 9 broader themes encompassing these codes. Code and theme descriptions, examples, and frequency percentages are found in Table 2. On average, coded text segments were 23.75 words (SD = 32.22 words, ranging from 1 to 344 words per segment).

### 3.1. Therapist Training Experiences

During the qualitative interview, therapists were asked about the strengths and weaknesses of the PCIT training they had received. All 34 therapists interviewed mentioned training strengths, which were classified into several themes identified via thematic analysis: experiential training components (e.g., role-plays, volunteer family; endorsed by 71% of therapists), logistics and organization of training (68%), trainer-related strengths (59%), and thoroughness of the information provided (56%). Several areas of weakness were identified by 65% of therapists across three themes: dislike of training location/format (endorsed by 65%), desire for more experiential learning (32%), and desire for more PDI-focused training (14%). Many therapists described the impact of the COVID-19 pandemic as having a negative impact on their training experience. For example, a therapist trained completely via Zoom recalled difficulties “*learning the skills [while] on camera …there’s a kid, a parent, and a therapist trying to show the toys and angling the camera so the therapist can coach the parent playing with the child. That was a little awkward, but that’s the only thing I think I would have preferred to have done differently*”. Another therapist recounted challenges of practicing a time-out “*with people you don’t know very well … it’s awkward because I am pretending to lift you up and you’re on a different screen so that was a bit clunky, which is a fault of the Zoom format*”.

Furthermore, all but one therapist mentioned strengths of the year-long consultation series, including the ability to receive experienced trainer advice on their own cases (76%), the group-based and collaborative nature (53%), and the experiential learning components (21%). Several weaknesses or areas for improvement related to the consultation call series were discussed during therapist interviews concerning the format of calls (i.e., virtual format; 24%), lack of PCIT cases (18%), inconvenient time (18%), and desiring more consultation call time (either more frequent calls or longer calls at the same frequency; 18%).

### 3.2. Shift to Telehealth Delivery of PCIT during the COVID-19 Pandemic

Almost all therapists (91%) identified obstacles to implementing PCIT via telehealth with their clients. The majority of therapists identified difficulty connecting with the family that was exacerbated by limited access to technology and other resources to support telehealth. For example, a therapist stated that many “*parents didn’t have a webcam, so I’m trying to do CDI on the phone. I can’t even see them. It’s like, who does CDI without seeing [the family]?*” Many therapists described families as being less comfortable with technology, which required additional time during each session spent troubleshooting technical difficulties. Almost half of the therapists (44%) cited technical difficulties occurring during every interaction with families. Furthermore, 18% of therapists reported that many families had poor telephone connection and did not have the opportunity to attempt a video call for treatment. One therapist described how “*on these windy days or rainy days [with] people that are rural or up on a mountain, [the connection] really does interfere [with therapy]*,” while another described difficulties “*in terms of basic connectivity issues, like the phone is dropping out because they don’t have phone service in their holler. I can very rarely get my PCIT families on video conference calls as it is*”. Moreover, when asked to describe factors that impacted their confidence when delivering therapy via telehealth, almost 80% of therapists described the unpredictable nature of PCIT sessions (e.g., extremely difficult child behavior, caregiver emotional outbursts) as a main reason for lacking confidence. Only 38% of therapists mentioned direct support from their agency during the transition to telehealth PCIT sessions, with another 5% of therapists commenting directly on a lack of support from their agency in conducting telehealth sessions.

While telehealth posed many challenges for therapists and families, over half of therapists (56%) interviewed described telehealth as being more convenient for families. Therapists described telehealth as a way for families who might not have received services otherwise due to living in a rural area or families who had difficulties “*fitting therapy into their schedule and [the therapist] would rather them do telehealth than no therapy at all*”. Many therapists also spoke to the increased generalizability of PCIT via telehealth, as they could “*make some of those coaching critiques and adjustments in [the child’s] regular daily environment, [which was] such an asset*”. One therapist even posited the ability for telehealth to move families along “*more quickly because we are removing the transition point of ‘Ok, now go home and try this on your own without me there to help you do it*’”.

### 3.3. Families Treated through the SOR Grant

In total, the 34 therapists initiated PCIT services with a total of 331 families (M = 10.03, SD = 12.79; this number includes families that completed at least an intake for PCIT services). Due to time constraints and therapist access to client data, therapists provided qualitative information on 147 of these cases during the interview process. While many cases had multiple referral reasons, the two most commonly indicated were concerns related to disruptive behaviors (81% of cases) and trauma (e.g., history of child maltreatment; 21% of cases). Moreover, to therapists’ knowledge, 21% of cases were directly involved with the court system at the time of starting treatment. Children’s guardians represented a wide variety of caregiving roles, including biological parents (68%), grandparents (14%), other relatives (12%), adoptive parents (9%), and foster parents (8%). Additional characteristics are presented in Table 3.

### 3.4. Impact of the Opioid Crisis on Families

Therapists described the impact of caregiver substance use and the opioid crisis on their PCIT cases in a variety of ways. Overall, therapists discussed a biological parent’s current or past opioid use in almost half of their PCIT cases (45%) and 10% of cases had opioid use in other members of the immediate family. Thus, children were often in the care of a biological parent with a history of opioid use or in the care of another family member (e.g., grandparent) due to parental substance use. For example, one therapist recounted how a child client’s “*biological mother overdosed and died… [while the child’s] biological father was a user too… so the grandmother became the caregiver of the child… [Parental drug use] was kind of the reason he was [in therapy]*”. During one interview, a therapist reflected on a grandmother who brought in her grandson for treatment. Before beginning PCIT, this child “*watched his father die of overdose [and then] watched the grandmother try to revive him through CPR*”. Another therapist described one case in which the biological mother “*was using opioids in addition to other substances and the child basically escaped the house on his own at nighttime and was walking along the streets so the adoptive parents took him in*”, highlighting the challenging interplay between difficult child behaviors and the effects of parental substance use. A plethora of other adverse experiences were described in the majority of cases (76%), which were classified into several themes, including instability in school or childcare (20% of cases), parental death or absence (19%), marital conflict or divorce (18%), financial problems/job insecurity (14%), and other negative experiences (e.g., homelessness; 38%).

Furthermore, therapists reflected on the ways in which opioid-impacted cases differed from their other PCIT cases. One key theme (endorsed by 21% of therapists) was that the caregivers in these cases differed from their typical case, and often required more from the therapist. Several therapists described differences working with grandparent caregivers, such as “*physical limitations of course are one thing, but also the differences in… generational beliefs about some of the core components that are built into PCIT and trying to sell [those components] to them [was] more difficult*”. Two additional themes reflected frequent crises (endorsed by 47% of therapists) and low family resources (endorsed by 18%), including “*a lot of disruption and sometimes it was just because basic needs aren’t met or transportation would be an issue or deciding whether to spend the time and money coming to treatment or finding their next meal for their family or something like that*”. Another therapist stated that “*challenges with resources related to food and utilities… were pretty common so sometimes they didn’t have electricity. From a telehealth perspective, there were challenges related to, not necessarily internet service because most of them lived someplace where they had [internet], not all of them, but most of them, but they would be out of minutes on their phone or … or their tablet got broken and… they didn’t have the resources to fix those issues quickly*”. Family opioid and other substance use also had a direct impact on therapists’ delivery of PCIT. Around 21% of therapists mentioned high rates of inconsistent attendance within opioid impacted cases. Children and families often wanted to discuss ongoing challenges related to absent family members, unpredictable and unstable living situations, and even homelessness. During one PCIT session, a therapist recalled navigating a challenging discussion when a child client, completing PCIT with his grandmother, asked “‘*When am I going to be able to see [my mother] again? What is she not doing right? Why doesn’t she want to see me?’... and there were allegations … that his biological father was physically abusive … so [the child] would say things like ‘My mom wouldn’t be this way if it wasn’t for my dad’ and he was trying to figure out some logical reasoning for something that really doesn’t have any logical explanation*”.

## 4. Discussion

To meet the needs of young children impacted by the opioid crisis in West Virginia, a rural state characterized by limited availability of evidence-based mental health services, the SOR grant was implemented to disseminate PCIT across the state. Through the SOR grant, 80 therapists were successfully trained in PCIT. However, several adjustments were made due to the onset of the COVID-19 pandemic during the completion of the grant. The current study used a qualitative content analysis approach to explore the role that the opioid epidemic and the COVID-19 pandemic played on the state-wide training of PCIT therapists across West Virginia. Overall, the aims of the SOR grant to increase availability and sustainability of PCIT in the state of West Virginia were met. As evidenced by the high training completion rates and high rates of therapist confidence with the protocol, there has been a notable expansion of the PCIT therapist and trainer workforce within the state of West Virginia. The majority of therapists endorsed unique aspects of PCIT training as strengths, including the involvement of a volunteer family that received intensive therapeutic services over the course of the initial 40 h PCIT training. Further, it is possible that pivoting to virtual delivery of PCIT training during the COVID-19 pandemic increased training convenience, accessibility, and satisfaction for therapists. Moreover, therapist engagement in the year-long consultation series was recounted with high levels of satisfaction.

It should be noted that while all therapists discussed strengths of and satisfaction with the training, many therapists preferred non-virtual formats. While training was described as thorough, well-organized, and hands-on regardless of the delivery format, this preference for in-person training persisted. It is possible that therapists who received training in a virtual format were less engaged with the experience, which may also explain the low response rate for those therapists compared to therapists who received their training in person. Findings highlighted unique challenges for PCIT training and implementation progressed in West Virginia, which provides insight into the barriers associated with implementing evidence-based treatments in similar rural regions of the United States. Thus, the current research adds to the growing body of literature that informs large-scale dissemination and implementation of treatments to address prevalent and costly early childhood behavioral needs [39,40,41,42].

Despite successes during the training year, such as high attendance rates at training and consultation calls and high satisfaction with training procedures, SOR therapists demonstrated low PCIT certification rates. This theme emerged among several interviews as almost one-fourth of therapists recalled challenges obtaining and carrying out PCIT cases in their agency. This challenge in securing PCIT cases set the stage for many therapists when describing areas for improvement in the consultation call series, as not all therapists had active cases to discuss with their respective trainer. At the time of data collection, only about one third of participants had obtained certification through PCIT International. While rates of certification are often not reported in other studies, some previous researchers have reported similarly low rates of PCIT therapist certification, with rates ranging from 0% [43] to 26.72% [28]. This further emphasizes the barriers that community clinicians face when attempting to become certified in PCIT, including staffing turnover and difficulty completing two full PCIT cases [43]. However, this outcome may improve over time as therapists from the current study can continue to work toward PCIT certification by participating in additional consultation calls and successfully graduating families from treatment.

Findings suggest that while many therapists trained through the SOR grant expanded their therapeutic repertoire to include PCIT telehealth services during the COVID-19 pandemic, this did not always translate to service delivery. Therapists in the current study highlighted the unique barriers faced by families in West Virginia that made it particularly challenging to access PCIT telehealth services. Notably, West Virginia is one of only five states that demonstrates declining rates of residents with access to high-speed internet in previous years [44]. While other states may have had greater capabilities to seamlessly leverage telehealth delivery of treatment [45], almost all of the therapists from the current study described frequent challenges to delivering PCIT via telehealth. For some, it was not possible to attempt this modality due to the technological limitations of the population served, as families often did not have access to a webcam, reliable internet, or even reliable phone service. Families in West Virginia face unique challenges in regard to the mountainous topography of Appalachia. Individuals living in rural, mountainous areas of Appalachia often are isolated from mental health care providers and experience greater challenges with internet speeds areas due to the physical terrain of the region [46]. Within the current sample, this may have led to greater difficulty in achieving the PCIT International certification criteria requiring the completion of two PCIT cases, which is expected to occur within the span of the year-long consultation series. Many researchers have highlighted successes in telemedicine during the COVID-19 pandemic [17,30]; however, other studies have documented the significant challenges faced by rural communities and providers when accessing and delivering therapeutic care via telehealth during the pandemic [18,19]. The current study contributes to this small body of literature by outlining the difficulty that PCIT providers in West Virginia experienced in reaching families during a time marked by elevated parental stress [47] and increased risk of harsh parenting practices [48]. Because PCIT significantly reduces parenting stress [49] and teaches caregivers to use positive parenting behaviors [50] in lieu of harsh parenting practices, the barriers to remote delivery during the height of the COVID-19 pandemic was particularly unfortunate. Additionally, the timing of the COVID-19 crisis was disruptive to the training of therapists who experienced difficulty completing the required two cases given the challenges related to technological infrastructure. Thus, the dissemination of PCIT in a region with limited technological infrastructure merits further attention and innovation.

Relatedly, the findings from the present study suggest that many of the families in treatment were directly or indirectly affected by the opioid epidemic, which contributed to engagement difficulties. Further, substance use and overdoses have risen over the course of the COVID-19 pandemic [51], potentially interacting with the challenge of navigating challenging child behaviors and the time required to engage in an involved behavioral parent training program. Themes discussed by therapists in describing their opioid-impacted caseload included frequent crises, inconsistent attendance in treatment, and strained family relationships. Because PCIT is a family therapy that necessitates both caregiver and child involvement in weekly sessions, it is possible that engagement in this treatment modality was especially challenging for families affected by the opioid crisis. This is troubling from a dissemination perspective, as PCIT could help address child behavioral and emotional problems [52] and traumatic stress [53] secondary to the opioid epidemic if families were able to engage in treatment. Therefore, this study highlights the urgent need for additional research in the area of PCIT dissemination in communities that have been impacted by the opioid crisis.

### 4.1. Strengths

The current study has many strengths that contribute to the field of implementation science. First, the qualitative approach allowed for a deeper understanding of the training and clinical implementation of PCIT across the state of West Virginia. Furthermore, this study incorporated perspectives from therapists in many diverse clinical settings, including community-based mental health clinics and child advocacy centers, which provided insight into barriers and facilitators of implementation in real-world settings. Moreover, therapists in the current study reached families that are not often represented in research [54]. For example, therapists trained through the SOR grant reported accepting many different client payment options that allowed for families with a range of financial resources to receive evidence-based mental health care. Including underrepresented populations was a strength of this study because it increases understanding of the feasibility and acceptability of telehealth-based PCIT within a sample of rural families greatly impacted by the opioid epidemic on many levels. As PCIT has been shown to reduce future risk of child maltreatment [55], the current study includes a large number of therapists who are serving families impacted by trauma. Efforts to disseminate PCIT described in the current study are essential when attempting to reach the most vulnerable children during the COVID-19 pandemic, a period characterized by increased caregiver stress and risk for child maltreatment [56]. The current study adds to the literature about barriers and faciliators when utilizing remote delivery of evidence-based behavioral parent training programs, such as PCIT.

### 4.2. Limitations

Several limitations are present. First, this study employed a retrospective, rather than controlled, research design. The 34 therapists who provided qualitative data about their experiences with PCIT delivery did so voluntarily. As such, it is possible that reporter bias is present within the current study. Specifically, study therapists sought out PCIT training and agreed to provide additional information about their experiences and thus, may have already had a more favorable view of the therapy than those who did not elect to participate. This subset of therapists may not be completely representative of all therapists who received training or therapists who receive PCIT or other treatment modality training in general. While many procedures were put in place to ensure methodical thematic analysis [57], the research team manually coded qualitative data provided by therapists, thus limiting the use of other analytic methods to examine data (e.g., co-occurrences between themes). Furthermore, the current study was conducted in rural Appalachia with a predominantly White sample, which may limit the generalizability of findings to non-Appalachian rural areas, non-rural areas, and areas characterized by greater racial and ethnic diversity. The qualitative data from families who received services were not available due to logistical constraints, thus limiting the ability to draw certain conclusions about PCIT implementation within the sample.

### 4.3. Future Directions

The current study highlights several important considerations for future researchers interested in the dissemination of PCIT to vulnerable populations. There remains a dearth of research on outcomes of PCIT among children with prenatal opioid exposure. While Egan and colleagues [58] recently explored the immediate outcomes of a community-based implementation of PCIT for children with prenatal substance exposure, there is a growing need for thorough and long-term investigations of parent- and child-level PCIT outcomes. Recently, Gurwitch and Warner-Metzger [59] proposed an adaptation to PCIT that aims to standardize the approach to addressing childhood trauma within the PCIT model. With families highly impacted by the opioid crisis, such as the current sample, it may be beneficial to incorporate the novel Trauma-Directed Interaction into future PCIT dissemination efforts to address trauma related to caregiver opioid use.

For future state-wide dissemination efforts, a hybrid dissemination and implementation model, one that employs both virtual and in-person training elements, may offer a necessary balance of efficiency and increased accessibility, while also cultivating high levels of satisfaction and connection among therapist trainees. The current PCIT training structure typically requires the following: (1) at least 40 h of face-to-face training or mentored co-therapy with a certified trainer, (2) attendance at consultation calls twice monthly, and (3) live observation or video review of therapist skills by a certified trainer [60]. Prior to the pandemic, the majority of the initial trainings or co-therapy mentorship occurred in person, which precluded many therapists from accessing such trainings due to geographical and/or financial constraints. A hybrid training model would be highly conducive to PCIT’s rigorous training structure in that it would increase therapists’ access to the initial trainings, particularly for those who live in regions with a scarcity of certified PCIT trainers. Further, a hybrid training model may offer a unique training experience that allows therapists to build competencies in delivering both in-person and virtual PCIT, which may mirror treatment delivery through the pandemic and beyond [31]. Many therapists described telehealth as a convenient way for families to access therapeutic services while also increasing generalization and application of PCIT skills. Future PCIT research should evaluate a hybrid PCIT model to determine if this provides an optimal method of skill development and application, potentially leading to greater improvements in child behavior. Researchers should also explore factors that facilitate the dissemination and implementation of evidence-based early interventions in rural populations as the current literature often neglects rural and historically underserved populations. Factors that may support expanded access to health care in urban settings may not generalize to rural populations, especially rural Appalachian populations, highlighting a critical area for additional research.

To address low engagement among a sample of families both directly and indirectly impacted by opioid use, future researchers may explore the implementation of PCIT with parents who are receiving intensive substance use recovery services, such as residential treatment. It is possible that the incorporating PCIT into the milieu of services for parents with substance use disorders may increase engagement and attainment of positive parenting strategies.

## 5. Conclusions

The current study revealed important lessons learned for future statewide PCIT dissemination efforts, especially within a state navigating challenges related to technology, substance use, and limited access to healthcare. Despite limitations, the current study expands upon previous literature examining the statewide dissemination of early childhood interventions, as challenges experienced at the training and therapist level are not well documented in the literature. Moreover, the study employs a qualitative approach that incorporates interview data to gain an in-depth understanding of the adversities that therapists face when learning and implementing a novel intervention in a rural region characterized by significant financial and environmental hardships.

## Figures and Tables

**Table 1:** Characteristics of Therapist Participants (*n* = 34).

Characteristic	*n* (%)
Credentials	
Doctoral	13 (38%)
Masters	20 (59%)
Student	2 (6%)
Licensed	26 (76%)
Years in profession (M = 10.47 years, SD = 8.03)	
0–10 years	19 (56%)
11–20 years	9 (26%)
21–30 years	4 (12%)
31–40 years	1 (3%)
Not reported	2 (6%)
Therapist setting	
Academic medical center	1 (3%)
Child advocacy center	5 (15%)
Community-based mental health center	7 (21%)
Early intervention	1 (3%)
Local health department	1 (3%)
Outpatient behavioral health clinic	11 (32%)
Private practice	6 (18%)
School-based mental health	1 (3%)
University-based training clinic	5 (15%)
Other	5 (15%)
Client payment format	
Insurance	25 (74%)
Self-pay	14 (41%)
Sliding-scale fee	13 (38%)
Services are free of charge	14 (41%)
Not reported	1 (3%)

**Table 2:** Qualitative Themes Endorsed by Therapists (*n* = 34).

Code Theme	Theme Specification	Example Excerpt	% of Therapists Endorsed
**Themes Related to Therapist Training Experiences**			
**Training Strengths** *Positive comments about or satisfaction with overall PCIT training*	**Experiential Components** *Positive comments about or satisfaction with role-play or volunteer family components of training*	“I think practicing, so doing our own practice to reach the mastery criteria was really helpful.That way I knew what I am expecting of parents and how challenging it can be. But also, that just helps me to understan’d what I’m teaching as I’m going through the Teach sessions as well.”	71%
	**Trainer-related Strengths** *Positive comments that are inherent to the trainer or related to what the trainer has done; trainer expertise*	“[The trainer] was really familiar with the ins-and-outs of practicing and providing. She was able to answer a lot of those really nuts and bolts questions and’ it wasn’t just content she knew, she knew the implementation of the craft and that doesn’t always happen. Some’times it’s just someone teaching you something new without having practiced it for a long time.”	59%
	**Logistics and Organization** *Positive comments about the organization and format of the training (e.g., virtual or in-person format, group setting)*	“Being able to talk to colleagues about what they were doing through this process as well and how they were implementing PCIT.” “It was smaller. Most of the ones I’ve attended have been big conferences. And I really appreciated that, just more personal aspect, more hands-on aspect.”	68%
	**Thorough/Information** *Positive comments about the training being informative, training providing helpful information, the training being thorough/detailed*	“We really did a good job of going through each part of it. How to prepare for it, the paperwork, how to introduce clients, parents, and children to how it works. And actually go through these scenarios. Now that was a strength.” “I didn’t feel like I was missing anything by the end of the training. I felt fully prepared to kind of jump into the actual practice by the end of it.”	56%
**Training Weaknesses** *Comments about weaknesses or areas for improvement related to the overall PCIT training*	**Experiential Components** *Suggestions or dissatisfaction with role-play or volunteer family components of training*	“We would have done more, more role-play… It’s hard to know what issues you’re going to face just from taking in the information, so I think we would have done a little bit more, more role play.” “I feel like we had excellent support in that but it would have been nice to even have a follow-up with the same patients that we had seen in the first place.”	32%
	**Logistics and Format** *Suggestions or negative comments related to the location or format of the training (e.g., virtual or in-person format, group size)*	“More time in the lab with families. Maybe having a second or third family to work with so everybody got a little more time.” “The only thing I think, maybe, there were like a lot of people in our training. I wish there was a way to get it more scaled down. It was a lot harder to kind of get one on one time with the trainers. That’s a small complaint, nothing big.”	65%
	**PDI Training** *A weakness or improvement related to the PDI portion of the training (e.g., wanting more PDI training, dissatisfaction of PDI training component)*	“The second training was all about the PDI phase and I remembered wishing we could have had longer. We didn’t do too much PDI during the first five day training.” “I wish that we would have more training in PDI because that one you don’t get to very often, to be honest. You get CDI a lot, but you don’t get a lot of PDI. So having more training in the PDI would have been highly effective for future use.”	14%
**Consultation Call Strengths** *Positive comments about or satisfaction with the consultation call series*	**Expert Trainer Advice** *Positive comments about receiving consultation on their cases from a seasoned PCIT trainer (e.g., case-specific questions, difficult ethical questions)*	“[Trainer] is so educated, she’s the master so it’s wonderful to get her eyes and her ideas on my cases to prepare me for when I’m working with family and things come up.”	76%
	**Group-based Format** *Therapist found the group-based nature of the consultation calls to be helpful (e.g., collegiality, support from the group)*	“I think finding out how other people were implementing PCIT and being able to collaborate with peers throughout the state was very helpful.”	53%
	**Experiential Components** *Therapists found the skills practice portion of consultation calls to be helpful*	“Real life learning experience through the consultation calls.”	21%
**Consultation Call Weaknesses** *Comments about weaknesses or areas for improvement related to the overall consultation call series*	**Not Enough Cases** *Comments related to how a lack of PCIT cases made the consultation call experience less helpful for them*	“I think the first thing I’m thinking of is that a lot of the people on my consultation calls haven’t had cases and that’s just kind of a bummer because there’s maybe two or three of us who do talk and get to troubleshoot a lot of things, but some other people haven’t had any cases at all, so they don’t get to participate as much so I don’t know that anything could be done about that.”	18%
	**Format** *Weaknesses or suggestions regarding the format of the consultation calls (e.g., group size)*	“Having it as a call made it just too easy to throw a load of laundry in the washer or something like that, if you had to.” “Well in our consultation calls I think there was… 15 or 20 of us.”	24%
	**Inconvenient Time** *Comments about when the consultation calls occurred within the week and day*	“For my group, it worked best for everybody’s schedules but like for me I work more later day/evening… I wasn’t a fan of the 8:00 AM calls.” “The calls started to conflict with my schedule.”	18%
	**Desire for More Time** *Dissatisfaction about the lack of time to go through cases and topics in consultation calls*	“I think, perhaps, sometimes we ran out of time talking about really interesting cases”	18%
**Themes Related to Experiences with Telehealth Implementation of PCIT**			
**Telehealth Facilitators** *Comments related to factors that facilitated the use of telehealth- delivered PCIT*	**Convenience for Client** *Positive attributes about Telehealth in relation to convenience for families*	“I think the positive piece is that families feel more comfortable in their home and it also fits for families who have resource needs. It fits their schedule, availability, timeline a little bit better, which are positives.”	56%
	**Reduced Transportation Barriers** *Telehealth eliminated obstacles associated with transportation*	“I will continue to offer telehealth because we live in a very rural state, some people just aren’t close to one of our offices or don’t have transportation.” “I think it’s a really good option for a lot of families who have a hard time traveling or don’t have the means to.”	44%
	**Agency Support** *Comments related to the agency for which the therapist works providing the support and infrastructure necessary to offer telehealth services*	“We went straight to telehealth. I would say, maybe within a week or two. They did a really good job of helping us switch over quickly…” “Most of us already had access to webcams of some nature, whether it was in a laptop, a phone…if you didn’t have that, then they bought them for us…”	38%
	**Generalizability** *Comments related to telehealth aiding in the generalizability of therapy skills to the family’s home environment*	“I think sometimes it’s a little more realistic for families to practice these skills at home because that’s where the behaviors are happening.” “One thing that I really have appreciated about telehealth with PCIT is that you can see kind of where the parents are doing special time, you can see more of… what the home environment looks like which is really helpful to have that direct view instead of relying on their explanations of what things look like…”	32%
**Telehealth Barriers** *Comments related to factors that hindered telehealth treatment accessibility or implementation*	**Therapist-Family Connection** *Comments related to telehealth impairing the therapist’s ability to connect with families*	“I think I have less of a connection with the client. I mean the parent or the child with telehealth. And it’s not because I’m afraid of it or I haven’t gotten used to it, the connection is just not the same. Not as personal.” “Just because in my experience with telehealth it’s been harder to engage them [children] than it has been adults.”	65%
	**Technology** *Comments related to either the family or therapist not having the necessary technology to support telehealth sessions*	“I did not have any success delivering PDI sessions virtually, simply because of the internet capabilities or lack thereof of my patients.” “There are technical problems, there’s lagging, there’s volume issues with the technology… You might be in the middle of something really important with a client, and in person it wouldn’t be an issue, but all of a sudden something cuts out or ‘can you repeat that,’ it kind of just disrupts the flow of therapy.”	44%
	**Low Family Resources** *Comments related to telehealth treatment not being accessible or as successful as in-person treatment due to low family resources*	“They simply didn’t have the bandwidth to be video conferencing with me and for me to be coaching them using a bug in the ear.” “...Trying to figure out how to get a parent equipment that they might need like earbuds or headphones that we would have for them automatically in the clinic.”	9%
	**Lack of Agency Support** *Comments related to treatment not being accessable or as successful as in-person treatment due to lack of support from the therapist’s agency (e.g., not providing the appropriate platforms or supplies)*	“… And my agency tried to keep us doing therapy, but they weren’t prepared at all for telehealth sessions… essentially everyone was just kind of laid off and then brought back in September.” “It was basically, ‘Go figure this out’, ‘Go do this’, … and there was no ability to do that. You just had to keep going and figure [telehealth] out.”	5%
**PCIT Confidence** *Comments about factors that contributed to the therapist’s confidence in delivering the PCIT model*	**General Experience** *Comments related to high levels of therapist confidence due to their previous experience in the field*	“Because I feel like those are a lot of skills that I use in therapy anyway with children and adults so I feel pretty confident with skills that I’m trying to teach the parents.”	14%
	**PCIT-specific Experience** *Comments related to high levels of therapist confidence due to their experience providing PCIT*	“I feel really confident that I know the model and that I can provide high fidelity PCIT treatment, that I’ve studied it, that I’ve practiced it, that I’ve got good consultation and supervision. And I see the results in my cases.”	67%
	**Predictability and Control** *Comments related to high levels of therapist confidence due to the predictable and consistent nature of therapy or feelings of control over the session*	“I have the skills and I have the manual, and I have a curriculum that I could follow to be successful at sticking to the integrity of the curriculum.”	47%
**Lack of PCIT Confidence** *Comments about factors that contributed to the therapist’s lack of confidence in delivering the PCIT model*	**General Experience** *Comments related to decreased levels of therapist confidence due to their lack of experience in the field*	“I always have room to grow, I’m not perfect in everything and there’s always new things to learn. There’s always children that are gonna come in and throw your game off.”	15%
	**PCIT-specific Experience** *Comments related to decreased levels of therapist confidence due to their lack of experience providing PCIT*	“I just don’t know if I can effectively portray the PCIT curriculum to the clients and they can understand and get it.” “I’m not as comfortable with [PDI] delivery because I’ve had a lot of dropouts … after things get successful.”	71%
	**Unpredictability and Lack of Control** *Comments related to decreased levels of therapist confidence due to the unpredictable and inconsistent nature of therapy or lack of control over the session*	“Because of the PDI component and the lack of being able to really support a parent if they get into a difficult situation. It’s not like you can press the stop button or really assist them when you’re virtual. And you don’t have much control over the environment that they have.” “There is just a lack of control in PDI and not having eyes on a kiddo could potentially be dangerous and that is anxiety producing.”	79%
	**Poor Internet Connection** *Comments related to decreased levels of therapist confidence due to poor internet connectivity*	“I feel less confident because, you know, technology in that situation is a little bit trickier to navigate, and technology is always going to raise its ugly head at some point or another [such as] the internet cutting out.” “I did have a situation where the phone that they were using died in the middle of a time-out and I was like ‘ahh,’ but it just feels like more is needed of me and technology sometimes works great and sometimes doesn’t.”	18%
**Themes Related to the Impact of the Opioid Crisis on Therapist Delivery of PCIT**			
**Opioid Case Differences** *Comments about the ways in which PCIT cases with opioid use in the family differed from cases without opioid use in the family*	**Frequent Crises** *Comments related to frequent crises experienced by PCIT families impacted by family member opioid use*	“... and at the same time there was a lot of instability. The [grandparents who were the child’s caregivers] would come in one week and they would be confident that their child [who was the child’s mother] was going to rehab and then the next week that plan had kind of flipped upside down. So I would kind of say … there was a lot of instability or inconsistency in kind of what our plans were from week to week.” “a lot more instability as far as like placement and visits.”	47%
	**Inconsistent Attendance** *Comments about how cases affected by opioid use had more inconsistent attendance than those not affected by opioid use*	“Probably the commitment of the families, sometimes it was challenging to keep them committed if there was opioid issues in part of the treatment” “much, much more higher percentage of dropout and like length of time between sessions.”	21%
	**Strained Family Relationships** *Comments about how cases affected by opioid use had greater strain in family relationships than those not affected by opioid use*	“Providing more psychoeducation how if that client themselves weren’t struggling with addiction, how addiction impacts that individual and affects the whole family.”	21%
	**Low Family Resources** *Comments about how families impacted by opioid use were lower resourced than other families*	“I would say [these cases] became more complicated because some people who were in the midst of recovery had difficulty maintaining all the resources they needed.”	18%

Note. PCIT = Parent–Child Interaction Therapy, PDI = Parent-Directed Interaction. Quotes have been edited for length and clarity.

**Table 3:** Characteristics of Therapist Participants (*n* = 34).

Characteristic	*n* (%)
Child diagnoses ^1^	
*ADHD* ^2^	37 (25%)
*Anxiety*	8 (5%)
*Autism*	9 (6%)
*Cognitive/developmental delay*	20 (14%)
*CD/DMDD/ODD* ^3,4,5^	26 (18%)
Caregiver role involved in treatment ^1^	
*Adoptive parents*	13 (9%)
*Foster parents*	12 (8%)
*Biological parents*	100 (68%)
*Grandparents*	21 (14%)
*Other relatives*	18 (12%)
Single caregiver involved in treatment	88 (60%)
Multiple caregivers involved in treatment	59 (40%)

^1^ indicates that clients could have more than one category (e.g., more than one referral reason) so the sum of percentages is greater than 100%. ^2^ ADHD = attention-deficit/hyperactivity disorder. ^3^ CD = conduct disorder. ^4^ DMDD = disruptive mood dysregulation disorder. ^5^ ODD = oppositional defiant disorder.

## Data Availability

Data available on request due to restrictions (e.g., confidentiality) from the corresponding author.
